# Supplementary material for: Current water contact and Schistosoma mansoni infection have distinct determinants: a data-driven population-based study in rural Uganda
Source: Nat Commun. 2024 Nov 8;15:9530. doi: 10.1038/s41467-024-53519-4 (PMC11549081; doi:10.1038/s41467-024-53519-4)
Supplement: Supplementary file 1 — Supplementary Information [file 41467_2024_53519_MOESM1_ESM.pdf]

Supplement for: Current water contact and *Schistosoma mansoni* infection have distinct determinants: a data-driven population-based study in rural Uganda

Fabian Reitzug[1], Narcis B. Kabatereine[2], Anatol M. Byaruhanga[2], Fred Besigye[2], Betty Nabatte[2], Goylette F. Chami[1*]

[1] Big Data Institute, Nuffield Department of Population Health, University of Oxford, Oxford, United Kingdom
[2] Division of Vector-Borne and Neglected Tropical Diseases Control, Uganda Ministry of Health, Kampala, Uganda

[*] Corresponding author
E-mail: goylette.chami@ndph.ox.ac.uk

Contents

1 Supplementary Tables **3**

2 Supplementary Figures **12**

List of Tables

S1 Variable definitions 3
S2 Key participant characteristics 8
S3 Water contact per activity 9
S4 Water contact frequency per activity (in trips per week) 9
S5 Water contact duration per activity (in hrs per week) 10
S6 Water contact at high-risk time per activity 10
S7 Comparison of variable selection between LRTs and BVS 11

List of Figures

S1 Distribution of water contact frequency and duration 12
S2 Distribution of water contact and participants over distance to water sites 13
S3 Variation in water contact over gender and age 14
S4 Model of water contact with occupation or gender removed 15
S5 Models of occupational, domestic, and recreational water contact 16
S6 Model of water contact frequency 17
S7 Model of water contact duration 18
S8 Variation in infection by water contact and age 19
S9 Variation in water contact and infection over waterbody distance 20
S10 Model of infection intensity 21
S11 Comparison of self-reported and direct observation data 22
S12 Sensitivity to infection outcome used 23
S13 Participant flow diagram 24
S14 Comparison of BAS and LRT results by variable types and levels 25

S15 Model of infection with additional snail variables 26
S16 Model of infection with additional water contact variables 27

1 Supplementary Tables

Table S1: Variable definitions

Variable	Definition	Type	Levels	Type of variable	Level of variable	Candidate variable for water contact	Candidate variable for infection
Age	Age to the nearest year	Continuous		Socio-demographic	Ind.-level	X	X
Age^2	Age to the nearest year squared	Continuous		Socio-demographic	Ind.-level	X	X
Female	Self-reported gender	Binary		Socio-demographic	Ind.-level	X	X
Education level attained	Highest level of education completed	Categorical	none, primary, secondary or higher	Socio-demographic	Ind.-level	X	X
Religion	Religion	Categorical	no religion/other, christian, muslim	Socio-demographic	Ind.-level	X	X
Enrolled at school	Participant currently enrolled (all participants aged 18+ coded as not enrolled)	Binary		Socio-demographic	Ind.-level	X	X
Domestic water contact	Participant engages in one or more of the following domestic water contact activities: getting drinking water, washing clothes with soap, washing clothes without soap, washing jerry cans or household items, washing clothes with soap	Binary		Water contact	Ind.-level	-	X
Occupational water contact	Participant engages in one or more of the following occupational water contact activities: collecting papyrus, fishing, fishmongering, collecting shells	Binary		Water contact	Ind.-level	-	X

Table S1: Variable definitions *(continued)*

Variable	Definition	Type	Levels	Type of variable	Level of variable	Candidate variable for water contact	Candidate variable for infection
Recreational water contact	Participant engages in one or more of the following recreational water contact activities: swimming or playing	Binary		Water contact	Ind.-level	-	X
Water contact frequency per week	Water contact frequency per individual per week across all activities (generated by combining the frequency of all individual activities)	Continuous		Water contact	Ind.-level	-	X
Water contact duration per week (hrs)	Water contact duration in hours per week across all activities (generated by combining frequency and duraction of all individual activities)	Categorical		Water contact	Ind.-level	-	X
Number of water contact activities	Number of distinct water contact activities a participant engaged in	Discrete		Water contact	Ind.-level	-	X
Occupation	Occupation of the household head (aged 18+), for all participants aged <18 occupation coded as ’none’	Categorical	none, fishing, fishmongering, farming, other	Socio-demographic	HH-level	X	X
Home owned, not rented	Household owns home, is not renting	Binary		Socio-demographic	HH-level	X	X

Table S1: Variable definitions *(continued)*

Variable	Definition	Type	Levels	Type of variable	Level of variable	Candidate variable for water contact	Candidate variable for infection
Home quality score	Quality of the roof, wall, and floor ranked from 1-4 and summed. The rank order of the materials was grass, sticks, plastic, and metal for the roof; mud and sticks, plastic, metal, and bricks or cement for the walls; mud, plastic, wood planks, and brick or cement for the floor.	Discrete		Socio-demographic	HH-level	X	X
People per HH	Number of people living in the household	Discrete		Socio-demographic	HH-level	X	X
HH has handwashing facility with soap	Household has fixed handwashing facility and soap	Binary		WASH	HH-level	X	X
Water available at place for handwashing	Water available at the place for handwashing	Binary		WASH	HH-level	X	X
HH purifies drinking water	Household uses any of the following methods of water purification: boil, add bleach, strain through cloth, use water filter, solar disinfect, wash jerry can with soap, or let stand and settle	Binary		WASH	HH-level	X	X
HH uses open freshwater source for drinking water	Household uses open freshwater source (swamp or lake) to collect drinking water	Binary		WASH	HH-level	X	X

Table S1: Variable definitions *(continued)*

Variable	Definition	Type	Levels	Type of variable	Level of variable	Candidate variable for water contact	Candidate variable for infection
Tap/borehole is main HH drinking water source	Tap/borehole is the main household drinking water source	Binary		WASH	HH-level	X	X
Home latrine	Household has home latrine (flush or pour flush toilet, covered pit latrine with/without privacy, or composting toilet)	Binary		WASH	HH-level	X	X
Faecal contamination at water site closest to HH	Contamination occurring at closest water site from the household, based on whether human stool was observed at the site	Binary		Environmental	Ind.-level	X	X
Distance to closest water site from HH (km)	Euclidian distance to the closest water site from household GPS location (in km)	Continuous		Environmental	HH-level	X	X
Distance to closest water site from village centre (km)	Euclidian distance from the household to the closest water site from village centre (in km)	basic		Environmental	Vill.-level	X	X
Distance to closest public latrine from HH (km)	Euclidian distance from the household to the closest public latrine (km)	Continuous		WASH	HH-level	X	X
Distance to closest public tap/borehole from HH (km)	Euclidian distance from the household to the closest public tap/borehole (km)	Continuous		WASH	HH-level	X	X
Type of water site closest to HH	Type of water site closest to the household	Categorical	river, marsh, beach, swamp, pond, other	Environmental	HH-level	X	X
Water site type in village	Type of water site present in the village	Categorical	none, landing site, beach	Environmental	Vill.-level	X	X

Table S1: Variable definitions *(continued)*

Variable	Definition	Type	Levels	Type of variable	Level of variable	Candidate variable for water contact	Candidate variable for infection
Number of water sites per village	Number of water sites per village	Discrete		Environmental	Vill.-level	X	X
Number of water sites within 1 km of HH	Number of sites within 1km of the household	Discrete		Environmental	HH-level	X	X
Closest water site to HH in village	Whether the closest water site to the household is within the village where the household resides	Binary		Environmental	HH-level	X	X
Village-level schistosomiasis prevalence (KK)	Village-level prevalence by Kato-Katz (moderate 10-49% prevalence, high>=50% prevalence)	Categorical	10-49%, 50+%	Biomedical	Vill.-level	X	X

Note:
Abbreviations: HH = household. Ind.-level = individual-level. Vill.-level = village-level. KK = Kato-Katz stool microscopy. Prop. = proportion. WASH = Water, sanitation, and hygiene.

Table S2: Key participant characteristics

Variable	No water contact (n= 1528)	Water contact (n= 1339)	P-value
Age (Mean ± SD)	21.3 ± 19.2	29.0 ± 15.9	<0.01
Age^2 (Mean ± SD)	821.3 ± 1356.0	1091.8 ± 1058.1	<0.01
Female (%)	798 (52.2)	775 (57.9)	<0.01
Education level attained (%)			<0.01
none	249 (16.3)	182 (13.6)	
primary	1179 (77.2)	1009 (75.4)	
secondary or higher	100 (6.5)	148 (11.1)	
Enrolled at school (%)	526 (34.4)	274 (20.5)	<0.01
Religion (%)			0.06
no religion/other	17 (1.1)	30 (2.2)	
christian	1268 (83.0)	1101 (82.2)	
muslim	243 (15.9)	208 (15.5)	
Number of water contact activities (median [IQR])	0.0 [0.0, 0.0]	1.0 [1.0, 2.0]	-
Water contact duration per week (hrs) (median [IQR])	0.0 [0.0, 0.0]	8.0 [3.5, 17.5]	-
Recreational water contact (%)	0 (0.0)	78 (5.8)	-
Domestic water contact (%)	0 (0.0)	969 (72.4)	-
Water contact frequency per week (median [IQR])	0.0 [0.0, 0.0]	6.0 [3.0, 11.0]	-
Occupational water contact (%)	0 (0.0)	472 (35.3)	-
Occupation (%)			<0.01
none	1113 (72.8)	604 (45.1)	
fishing	25 (1.6)	226 (16.9)	
fishmongering	20 (1.3)	88 (6.6)	
farming	225 (14.7)	285 (21.3)	
other	145 (9.5)	136 (10.2)	
Home owned, not rented (%)	1297 (84.9)	1129 (84.3)	0.71
HH has handwashing facility with soap (%)	173 (11.3)	110 (8.2)	0.01
Home latrine (%)	1326 (86.8)	1133 (84.6)	0.11
Tap/borehole is main HH drinking water source (%)	774 (50.7)	739 (55.2)	0.02
HH uses open freshwater source for drinking water (%)	277 (18.1)	278 (20.8)	0.08
HH purifies drinking water (%)	354 (23.2)	293 (21.9)	0.44
Water available at place for handwashing (%)	265 (17.3)	199 (14.9)	0.08
People per HH (Mean ± SD)	3.6 ± 1.3	3.6 ± 1.4	0.96
Home quality score (Mean ± SD)	5.6 ± 1.1	5.7 ± 1.1	0.19
Faecal contamination at water site closest to HH (%)	719 (47.1)	577 (43.1)	0.04
Distance to closest water site from HH (km) (Mean ± SD)	0.4 ± 0.4	0.3 ± 0.3	<0.01
Distance to closest water site from village centre (km) (Mean ± SD)	0.3 ± 0.3	0.3 ± 0.2	<0.01
Distance to closest public tap/borehole from HH (km) (Mean ± SD)	0.3 ± 0.3	0.3 ± 0.3	0.76
Distance to closest public latrine from HH (km) (Mean ± SD)	1.1 ± 1.4	1.0 ± 1.4	0.03
Type of water site closest to HH (%)			<0.01
river	188 (12.3)	93 (6.9)	
marsh	590 (38.6)	412 (30.8)	
beach	491 (32.1)	566 (42.3)	
swamp	161 (10.5)	149 (11.1)	
pond	86 (5.6)	113 (8.4)	
other	12 (0.8)	6 (0.4)	
Number of water sites per village (Mean ± SD)	3.7 ± 1.4	4.0 ± 1.5	<0.01

Table S2: Key participant characteristics *(continued)*

Variable	No water contact (n= 1528)	Water contact (n= 1339)	P-value
Water site type in village (%)			<0.01
none	850 (55.6)	704 (52.6)	
landing site	173 (11.3)	104 (7.8)	
beach	505 (33.0)	531 (39.7)	
Number of water sites within 1 km of HH (Mean ± SD)	1.2 ± 1.3	1.3 ± 1.3	<0.01
Closest water site to HH in village (%)	566 (37.0)	605 (45.2)	<0.01
Village-level schistosomiasis prevalence (KK) = 50+% (%)	594 (38.9)	518 (38.7)	0.95

Note:
For binary variables, number and proportions [N (%)] are shown and Chi-squared tests were used to test for group differences. For all normally distributed continuous variables, mean and standard deviation (mean +/- sd) are shown and two sample t-tests were performed to test for group differences. For all non-normally distributed variables, median and interquartile range (median [IQR]) are shown and Kruskal-Wallis Rank Sum Tests were performed to test for group differences. Abbreviations: HH = household. Prop. = proportion.

Table S3: Water contact per activity

Variable	Overall	Female	Male	P-value
Getting drinking water (%)	497 (17.3)	348 (22.1)	149 (11.5)	<0.001
Washing clothes with soap (%)	481 (16.8)	368 (23.4)	113 (8.7)	<0.001
Fishing (%)	354 (12.3)	39 (2.5)	315 (24.3)	<0.001
Washing clothes without soap (%)	152 (5.3)	115 (7.3)	37 (2.9)	<0.001
Washing jerry cans or other household items (%)	144 (5.0)	108 (6.9)	36 (2.8)	<0.001
Fishmongering (%)	104 (3.6)	90 (5.7)	14 (1.1)	<0.001
Swimming or playing (%)	78 (2.7)	32 (2.0)	46 (3.6)	0.018
Bathing with soap (%)	49 (1.7)	29 (1.8)	20 (1.5)	0.640
Bathing without soap (%)	38 (1.3)	19 (1.2)	19 (1.5)	0.658
Collecting papyrus (%)	16 (0.6)	8 (0.5)	8 (0.6)	0.888
Collecting shells (%)	15 (0.5)	11 (0.7)	4 (0.3)	0.238

Note:
Number and percentage of participants with water contact [n (%)] per activity. Chi-squared tests were used to test for group differences.

Table S4: Water contact frequency per activity (in trips per week)

Variable	Overall	Female	Male	P-value
Trips to water collecting shells (median [IQR])	5.0 [3.0, 7.0]	5.0 [2.5, 7.0]	7.0 [6.5, 7.0]	0.13
Trips to water swimming or playing (median [IQR])	5.0 [3.0, 7.0]	5.0 [3.0, 7.0]	6.0 [3.0, 7.0]	0.78
Trips to water collecting papyrus (median [IQR])	5.0 [3.0, 5.5]	4.5 [2.8, 5.0]	5.0 [3.0, 7.0]	0.33
Trips to water bathing with soap (median [IQR])	5.0 [2.0, 7.0]	6.0 [2.0, 7.0]	3.0 [2.0, 7.0]	0.12
Trips to water fishing (median [IQR])	4.0 [2.0, 7.0]	7.0 [3.0, 7.0]	4.0 [2.0, 7.0]	0.06
Trips to water fishmongering (median [IQR])	3.5 [1.0, 7.0]	4.0 [1.0, 7.0]	3.0 [1.2, 6.8]	0.62
Trips to water bathing without soap (median [IQR])	3.0 [2.0, 7.0]	3.0 [2.0, 7.0]	4.0 [2.5, 7.0]	0.37

Table S4: Water contact frequency per activity (in trips per week) *(continued)*

Variable	Overall	Female	Male	P-value
Trips to water washing jerry cans or other household items (median [IQR])	3.0 [2.0, 7.0]	3.0 [2.0, 7.0]	3.0 [2.0, 7.0]	0.97
Trips to water washing clothes without soap (median [IQR])	3.0 [2.0, 6.0]	3.0 [2.0, 5.0]	4.0 [3.0, 10.0]	<0.01
Trips to water washing clothes with soap (median [IQR])	2.0 [2.0, 5.0]	2.0 [2.0, 5.0]	2.0 [1.0, 5.0]	1.00
Trips to water getting drinking water (median [IQR])	7.0 [5.0, 14.0]	7.0 [5.0, 14.0]	7.0 [5.0, 12.0]	0.84

Note:
Median and interquartile range (median [IQR]) are shown. Kruskal-Wallis Rank Sum Tests were performed to test for group differences.

Table S5: Water contact duration per activity (in hrs per week)

Variable	Overall	Female	Male	P-value
Time spent fishing (hrs) (median [IQR])	4.0 [3.0, 4.0]	4.0 [2.5, 4.0]	4.0 [3.0, 4.0]	0.81
Time spent collecting papyrus (hrs) (median [IQR])	3.0 [2.0, 3.0]	3.0 [3.0, 4.0]	2.0 [1.8, 3.0]	0.01
Time spent collecting shells (hrs) (median [IQR])	2.0 [2.0, 3.5]	3.0 [2.0, 4.0]	2.0 [2.0, 2.2]	0.33
Time spent fishmongering (hrs) (median [IQR])	2.0 [1.0, 3.0]	2.0 [1.0, 3.0]	2.0 [1.0, 3.0]	0.98
Time spent washing clothes without soap (hrs) (median [IQR])	2.0 [1.0, 3.0]	2.0 [1.0, 3.0]	2.0 [1.0, 2.0]	0.90
Time spent washing clothes with soap (hrs) (median [IQR])	2.0 [1.0, 2.0]	2.0 [1.0, 2.0]	2.0 [1.0, 3.0]	0.61
Time spent swimming or playing (hrs) (median [IQR])	1.0 [1.0, 2.0]	1.0 [0.5, 3.0]	1.0 [1.0, 2.0]	0.92
Time spent bathing without soap (hrs) (median [IQR])	1.0 [0.5, 2.0]	1.0 [0.8, 2.0]	1.0 [0.5, 2.0]	0.49
Time spent washing jerry cans or other household items (hrs) (median [IQR])	1.0 [0.5, 1.2]	1.0 [0.5, 1.2]	1.0 [1.0, 1.2]	0.53
Time spent getting drinking water (hrs) (median [IQR])	1.0 [0.5, 1.0]	1.0 [0.5, 1.0]	1.0 [0.5, 1.0]	0.54
Time spent bathing with soap (hrs) (median [IQR])	0.5 [0.5, 2.0]	0.5 [0.5, 2.0]	0.5 [0.5, 2.0]	0.90

Note:
Median and interquartile range (median [IQR]) are shown. Kruskal-Wallis Rank Sum Tests were performed to test for group differences.

Table S6: Water contact at high-risk time per activity

Variable	Overall	Female	Male	P-value
Washing clothes with soap at high-risk time (%)	167 (34.7)	124 (33.7)	43 (38.1)	0.460
Getting drinking water at high-risk time (%)	92 (18.5)	69 (19.8)	23 (15.4)	0.304
Washing jerry cans or other household items at high-risk time (%)	54 (37.5)	46 (42.6)	8 (22.2)	0.047
Fishing at high-risk time (%)	41 (11.6)	7 (17.9)	34 (10.8)	0.188
Washing clothes without soap at high-risk time (%)	35 (23.0)	26 (22.6)	9 (24.3)	1.000
Fishmongering at high-risk time (%)	21 (20.2)	21 (23.3)	0 (0.0)	0.067
Bathing with soap at high-risk time (%)	16 (32.7)	7 (24.1)	9 (45.0)	0.222
Swimming or playing at high-risk time (%)	13 (16.7)	5 (15.6)	8 (17.4)	1.000

Table S6: Water contact at high-risk time per activity *(continued)*

Variable	Overall	Female	Male	P-value
Bathing without soap at high-risk time (%)	12 (31.6)	5 (26.3)	7 (36.8)	0.727
Collecting shells at high-risk time (%)	5 (33.3)	3 (27.3)	2 (50.0)	0.560
Collecting papyrus at high-risk time (%)	4 (25.0)	3 (37.5)	1 (12.5)	0.569

Note:
Number and percentage of participants with water contact [n (%)] at high-risk time per activity. High-risk time was defined as water contact during peak cercarial shedding hours (10 am-3 pm). Chi-squared tests were used to test for group differences.

Table S7: Comparison of variable selection between LRTs and BVS

variable	Infection status (LRT)	Infection status (BAS)	Water contact (LRT)	Water contact (BAS)
Age	X	X	X	X
Age^2	X	X	X	X
Female	X	-	X	X
Education level attained	X	X	X	-
Enrolled at school	X	-	X	-
Occupational water contact	X	-	-	-
Number of water contact activities	X	-	-	-
Occupation	X	X	X	X
HH has handwashing facility with soap	X	-	-	-
Home latrine	X	-	-	-
Faecal contamination at water site closest to HH	-	-	X	X
Distance to closest water site from HH (km)	X	-	X	-
Distance to closest water site from village centre (km)	-	-	X	-
Distance to closest public latrine from HH (km)	X	-	-	X
Type of water site closest to HH	X	-	-	X
Water site type in village	-	X	-	-
Number of water sites per village	-	X	-	X
Closest water site to HH in village	-	-	X	X
Village-level schistosomiasis prevalence (KK)	-	X	-	X

Note:
Comparison of variable sets selected using likelihood ratio tests (LRTs) at pr <0.05 with the variable sets selected via Bayesian variable selection (BVS) with marginal inclusion probabilities p >= 0.5 (i.e., variables included in the median probability model).

2 Supplementary Figures

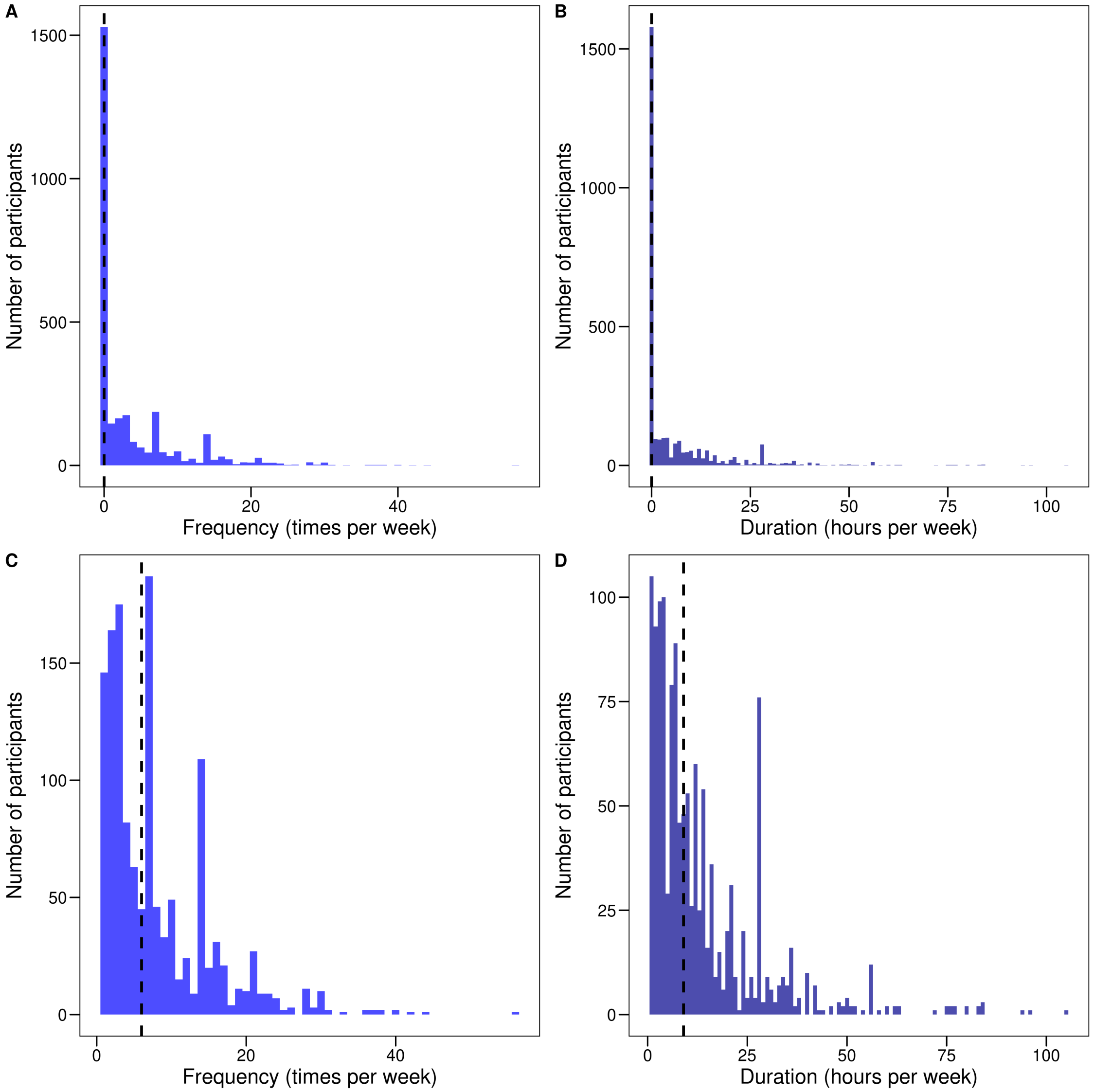

Figure S1: **A.** Distribution of water contact frequency among all participants. **B.** Distribution of water contact duration among all participants. **C.** Distribution of water contact frequency among participants with water contact only. **D.** Distribution of water contact duration among participants with water contact only. Dashed vertical line = median.

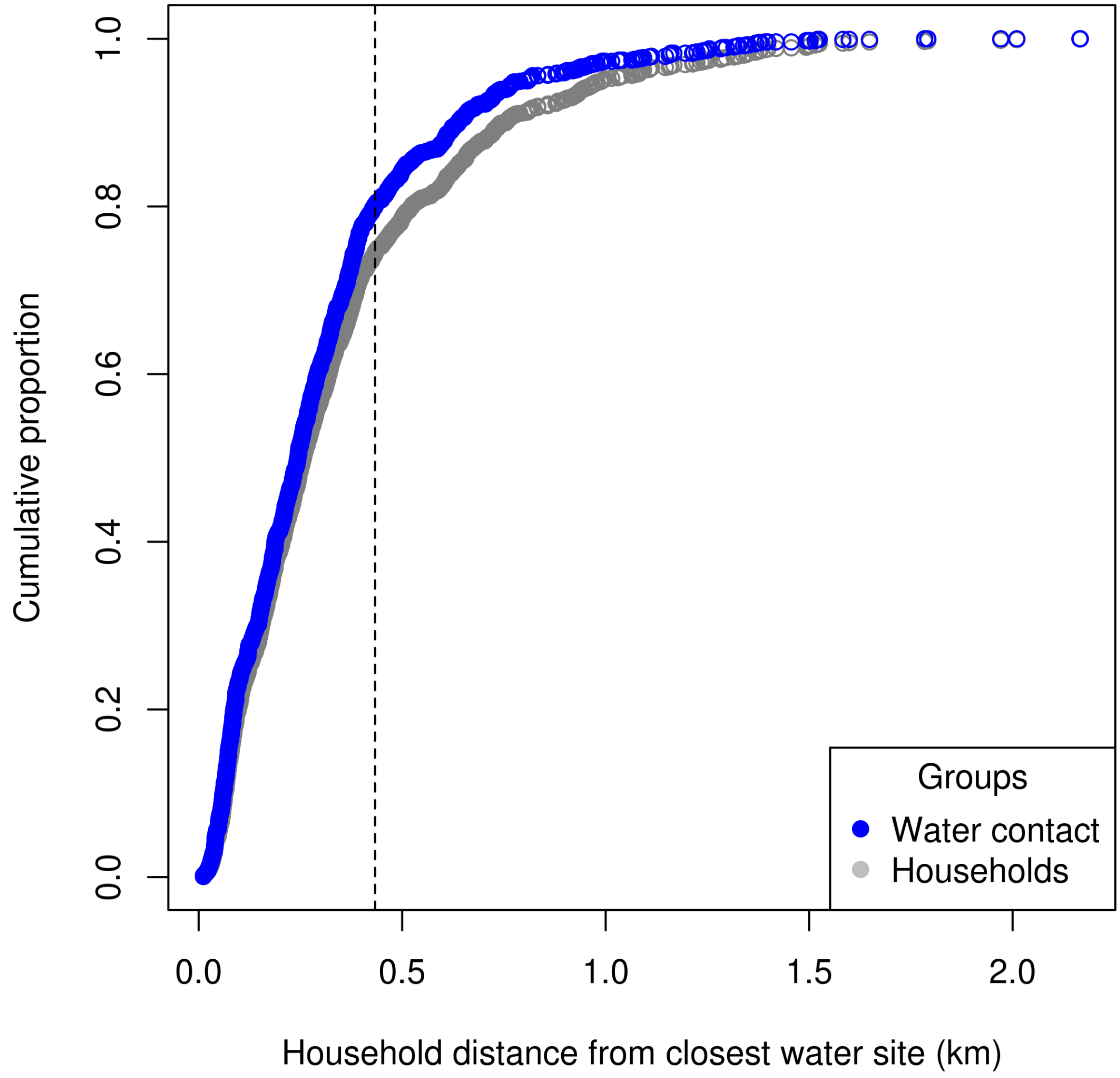

Figure S2: Cumulative proportion of all water contacts/participants by household distance to the closest water site (n=2867). Dashed line showing the distance threshold where 80% of all individuals with water contact lived which is within 0.43 km from the shoreline. By comparison 74.3% of the overall study population lived lived which is within 0.43 km from the shoreline.

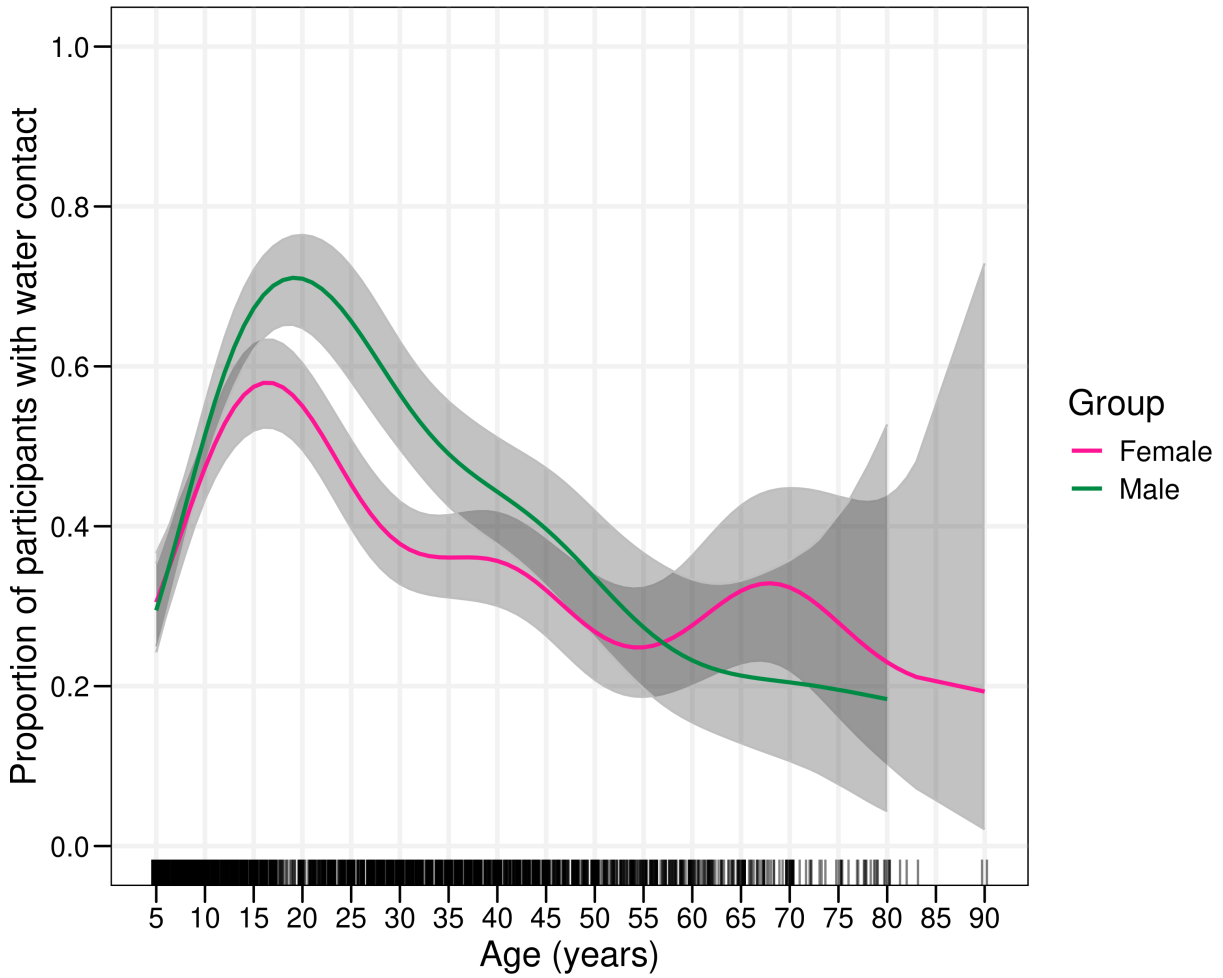

Figure S3: Generalised additive model predicting the proportion of participants with water contact over age and gender.

Variable	OR (95% CI) main model	OR (95% CI) occupation removed	OR (95% CI) gender removed
Age	1.18 (1.15 to 1.21)	1.19 (1.17 to 1.21)	1.18 (1.16 to 1.21)
Age^2	1.00 (1.00 to 1.00)	1.00 (1.00 to 1.00)	1.00 (1.00 to 1.00)
Female	1.41 (1.17 to 1.68)	1.05 (0.89 to 1.24)	
Occupation (ref=none)			
fishing	6.67 (4.06 to 10.95)		5.26 (3.26 to 8.49)
fishmongering	2.10 (1.12 to 3.95)		2.15 (1.15 to 4.01)
farming	0.90 (0.67 to 1.20)		0.90 (0.67 to 1.20)
other	0.48 (0.34 to 0.67)		0.46 (0.33 to 0.64)
Faecal contamination at water site closest to HH	0.76 (0.63 to 0.92)	0.80 (0.67 to 0.96)	0.77 (0.64 to 0.93)
Distance to closest public latrine from HH (km)	0.87 (0.81 to 0.94)	0.87 (0.81 to 0.93)	0.87 (0.81 to 0.94)
Type of water site closest to HH (ref=river)			
marsh	1.28 (0.93 to 1.78)	1.45 (1.05 to 1.99)	1.27 (0.92 to 1.76)
beach	2.49 (1.75 to 3.55)	2.73 (1.92 to 3.87)	2.52 (1.77 to 3.59)
swamp	1.57 (1.04 to 2.37)	1.90 (1.26 to 2.86)	1.60 (1.06 to 2.41)
pond	3.24 (2.05 to 5.13)	3.66 (2.32 to 5.76)	3.28 (2.08 to 5.18)
other	0.92 (0.30 to 2.78)	1.17 (0.40 to 3.43)	0.96 (0.32 to 2.84)
Number of water sites per village	1.13 (1.05 to 1.21)	1.13 (1.06 to 1.21)	1.13 (1.05 to 1.21)
Closest water site to HH in village	1.49 (1.25 to 1.78)	1.43 (1.20 to 1.71)	1.50 (1.26 to 1.80)
Village-level schistosomiasis prevalence (KK) (ref=10-49%)			
50+%	0.70 (0.57 to 0.87)	0.73 (0.60 to 0.90)	0.70 (0.56 to 0.86)
District (ref=Mayuge)			
Buliisa	1.02 (0.80 to 1.30)	1.14 (0.90 to 1.43)	1.02 (0.81 to 1.30)
Pakwach	0.96 (0.75 to 1.24)	1.09 (0.85 to 1.39)	0.94 (0.74 to 1.21)

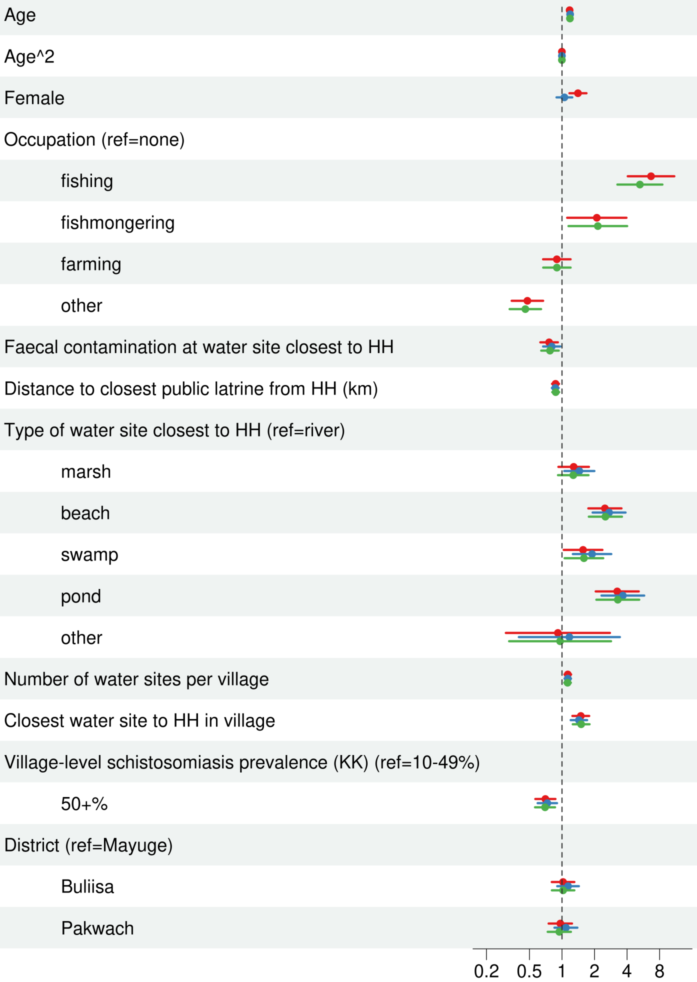

Figure S4: Logistic regression model predicting water contact (main model shown in Fig. 7) versus the same model with the occupation variable removed and gender removed, respectively (n=2867 for all models). Standard errors clustered at the household level. Abbreviations: OR = odds ratio. CI = confidence interval.

Variable	OR (95% CI) Occupational	OR (95% CI) Domestic	OR (95% CI) Recreational
Age	1.29 (1.21 to 1.37)	1.14 (1.11 to 1.17)	0.98 (0.90 to 1.07)
Age^2	1.00 (1.00 to 1.00)	1.00 (1.00 to 1.00)	1.00 (1.00 to 1.00)
Female	0.17 (0.11 to 0.27)	2.43 (1.97 to 3.00)	0.28 (0.11 to 0.73)
Occupation (ref=none)			
fishing	30.49 (15.18 to 61.24)	0.04 (0.01 to 0.09)	
fishmongering	16.48 (7.98 to 34.03)	0.25 (0.14 to 0.45)	
farming	1.42 (0.73 to 2.76)	0.93 (0.68 to 1.26)	
other	1.57 (0.80 to 3.11)	0.40 (0.28 to 0.58)	
Faecal contamination at water site closest to HH	0.84 (0.57 to 1.24)	0.75 (0.61 to 0.92)	0.69 (0.30 to 1.57)
Distance to closest public latrine from HH (km)	0.99 (0.86 to 1.15)	0.88 (0.82 to 0.96)	0.86 (0.64 to 1.16)
Type of water site closest to HH (ref=river)			
marsh	1.82 (0.79 to 4.18)	1.15 (0.78 to 1.68)	
beach	2.64 (1.08 to 6.47)	2.29 (1.53 to 3.43)	
swamp	4.52 (1.70 to 12.05)	1.15 (0.72 to 1.82)	
pond	2.86 (1.00 to 8.20)	2.16 (1.29 to 3.61)	
other	1.67 (0.43 to 6.56)	0.85 (0.26 to 2.79)	
Number of water sites per village	1.16 (1.01 to 1.34)	1.06 (0.98 to 1.16)	0.97 (0.68 to 1.36)
Closest water site to HH in village	0.86 (0.58 to 1.27)	1.61 (1.33 to 1.94)	2.69 (1.13 to 6.41)
Village-level schistosomiasis prevalence (KK) (ref=10-49%)			
50+%	0.56 (0.36 to 0.87)	0.76 (0.60 to 0.96)	1.12 (0.49 to 2.53)
District (ref=Mayuge)			
Buliisa	3.46 (2.04 to 5.89)	0.73 (0.56 to 0.95)	4.18 (1.34 to 13.07)
Pakwach	5.52 (3.25 to 9.36)	0.57 (0.43 to 0.74)	1.49 (0.45 to 4.91)

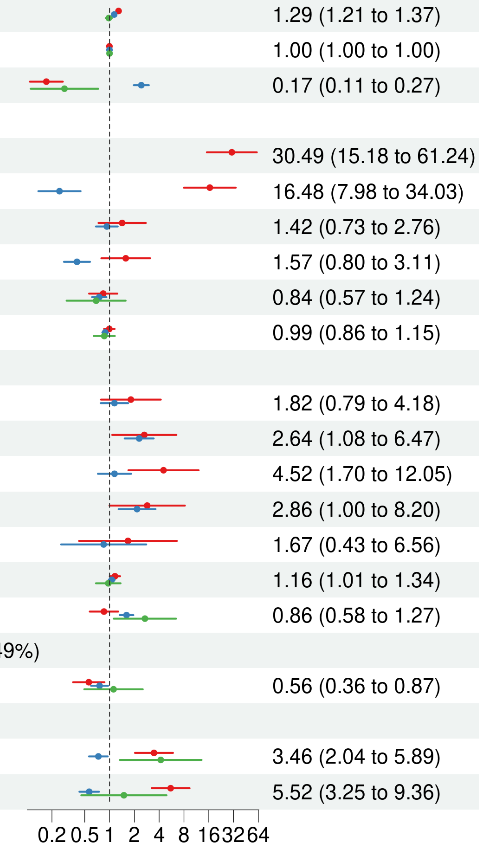

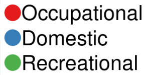

Figure S5: Results from seperate logistic regression models with standard errors clustered at the household level predicting domestic, occupational, and recreational water contact among participants that do not engage in multiple types of water contact (n=2684). As not all categories in the water site type and the occupation variables were sufficiently represented in the recreational water contact model, we removed these two variables when predicting recreational water contact. Abbreviations: OR = odds ratio. CI = confidence interval.

Variable	OR (95% CI)
Age	1.01 (1.00 to 1.02)
Age^2	1.00 (1.00 to 1.00)
Female	1.09 (0.97 to 1.22)
Occupation (ref=none)	
fishing	0.51 (0.43 to 0.61)
fishmongering	0.62 (0.49 to 0.77)
farming	0.55 (0.46 to 0.65)
other	0.63 (0.53 to 0.76)
Faecal contamination at water site closest to HH	1.02 (0.91 to 1.13)
Distance to closest public latrine from HH (km)	0.96 (0.93 to 1.00)
Type of water site closest to HH (ref=river)	
marsh	1.16 (0.93 to 1.44)
beach	1.23 (0.99 to 1.52)
swamp	1.20 (0.93 to 1.56)
pond	1.63 (1.24 to 2.13)
other	0.69 (0.28 to 1.65)
Number of water sites per village	1.07 (1.02 to 1.11)
Closest water site to HH in village	1.04 (0.95 to 1.14)
Village-level schistosomiasis prevalence (KK) (ref=10-49%)	
50+%	1.04 (0.93 to 1.15)
District (ref=Mayuge)	
Buliisa	0.68 (0.59 to 0.78)
Pakwach	0.84 (0.74 to 0.96)

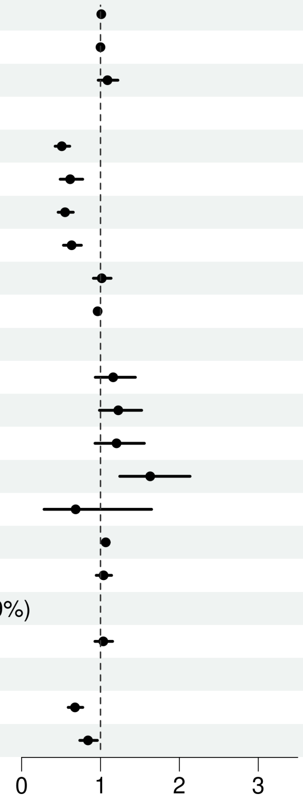

Figure S6: Negative binomial regression model predicting water contact frequency among all participants (n=2867). Standard errors clustered at the household level. A zero inflation test suggested that the predicted number of zeros (1440) was not significantly different from the number of observed zeros (1528), ratio=0.94, at the default tolerance of 0.1 of the `check_zeroinflation` function in the R package `performance`. Abbreviations: OR = odds ratio. CI = confidence interval.

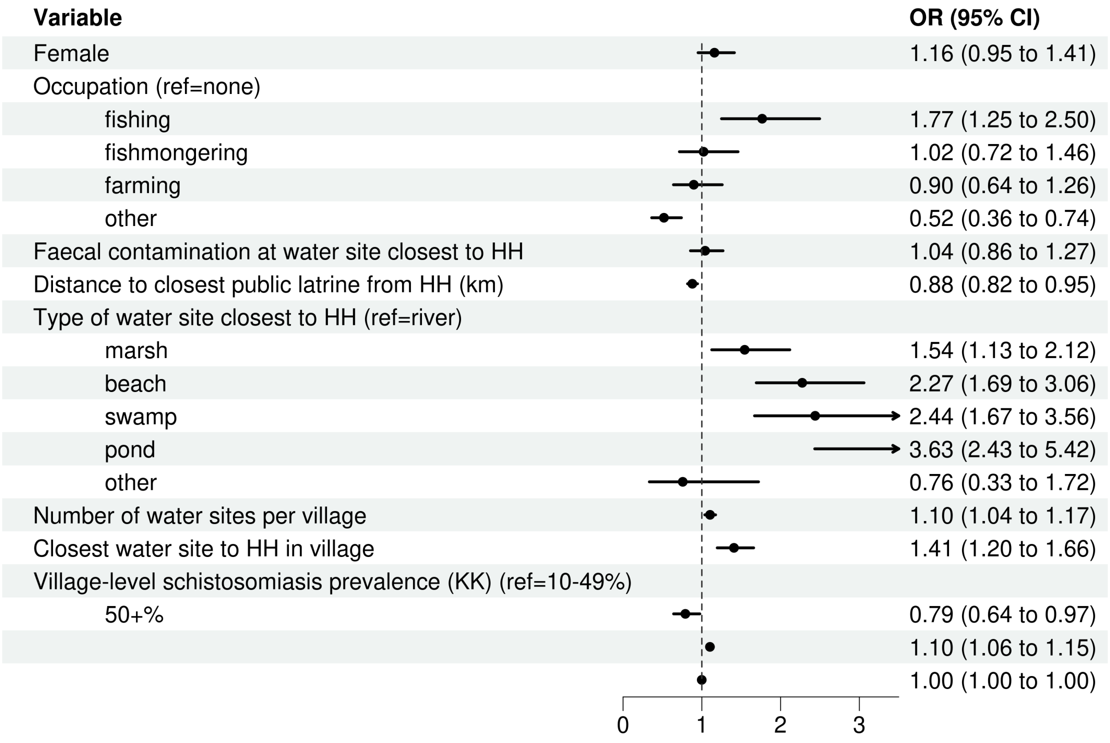

Figure S7: Negative binomial regression model predicting water contact duration among all participants (n=2867). Standard errors clustered at the household level. A zero inflation test suggested that the predicted number of zeros (1479) was not significantly different from the number of observed zeros (1568), ratio=0.94, at the default tolerance of 0.1 of the `check_zeroinflation` function in the R package `performance`. Abbreviations: OR = odds ratio. CI = confidence interval.

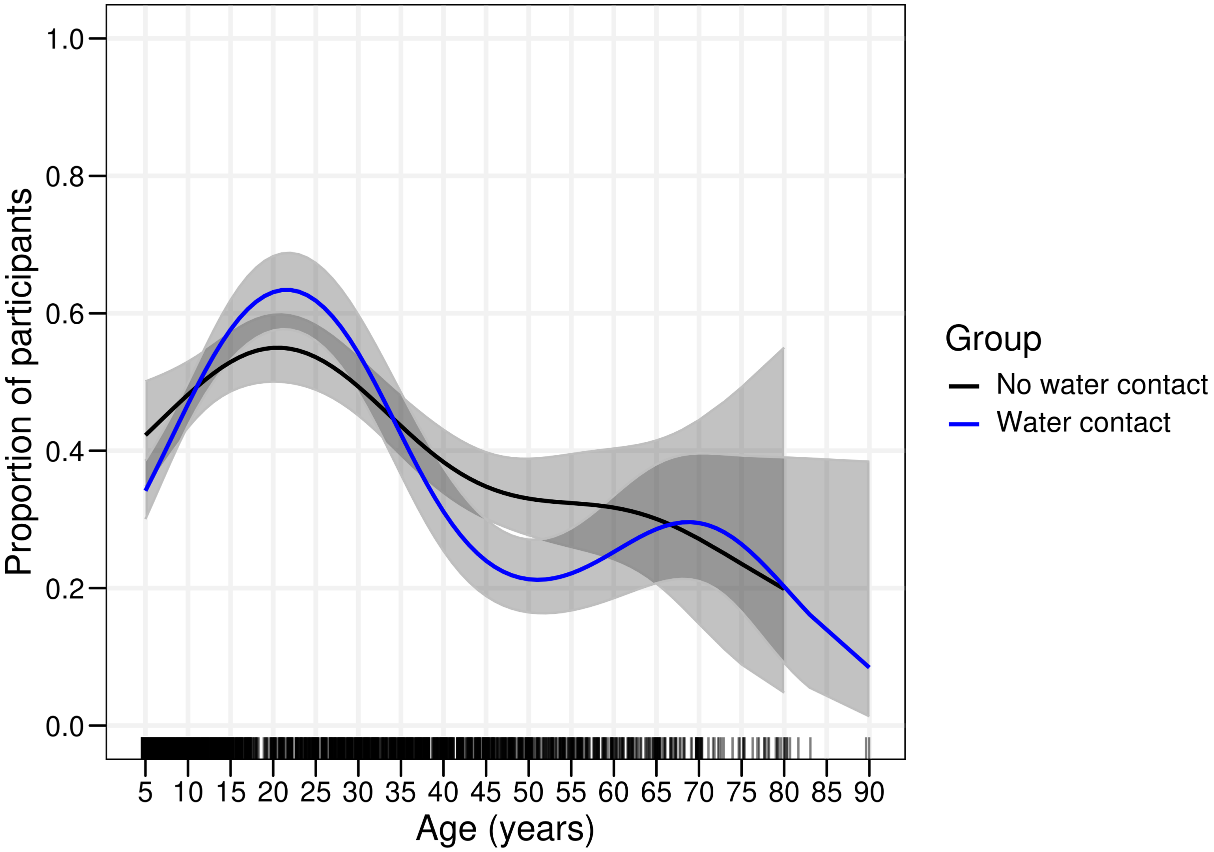

Figure S8: Generalised additive model predicting the proportion of participants with *S. mansoni* infection by water contact status and age (i.e., comparing infection outcomes in participants with current water contact vs participants without current water contact over age, n=2867).

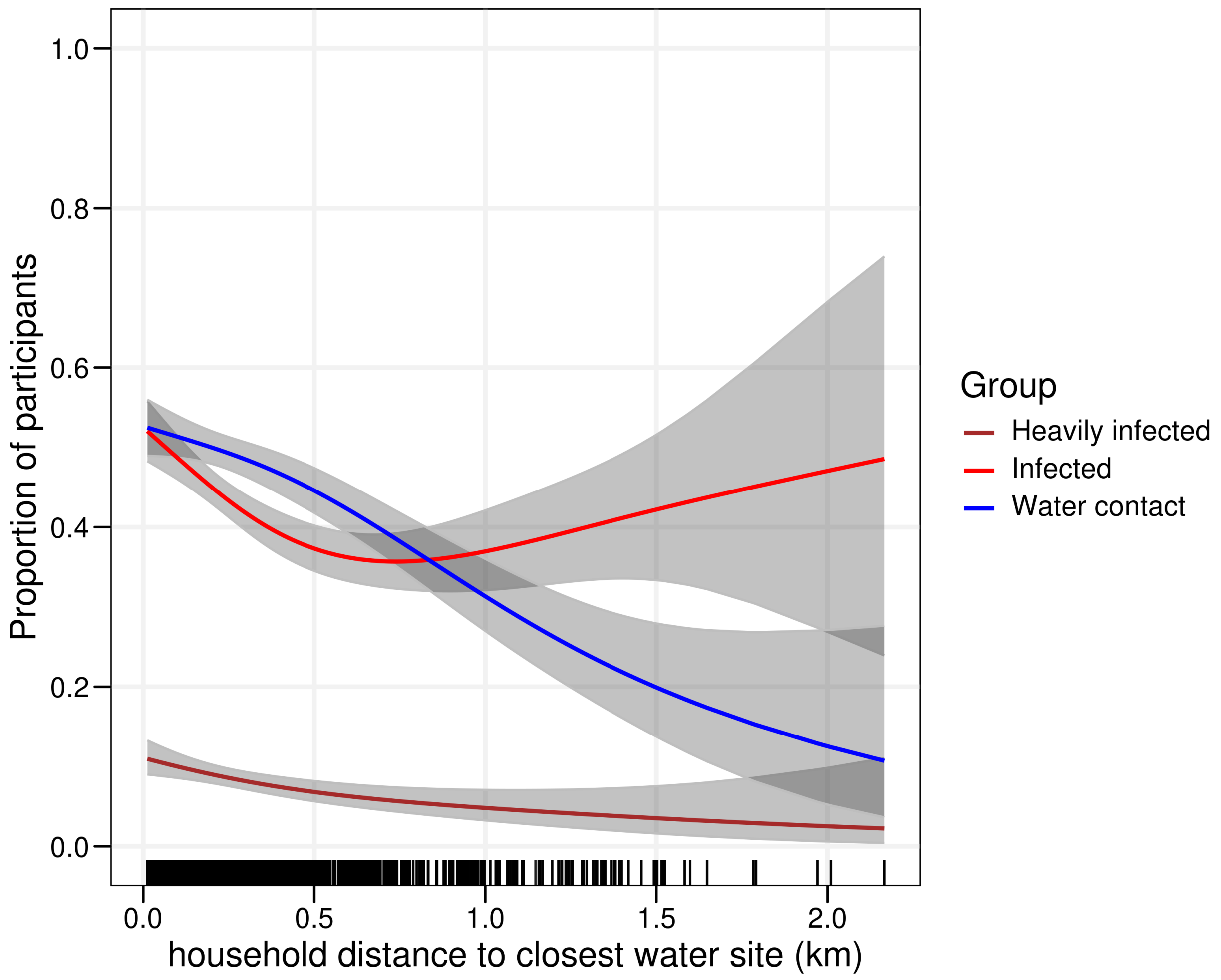

Figure S9: Generalised additive model predicting the proportion of participants with *S. mansoni* infection and heavy infection (400+ eggs per gram of stool, by Kato-Katz micrsocopy) as well as the proportion of participants with water contact over household distance to the closest water site (n=2867).

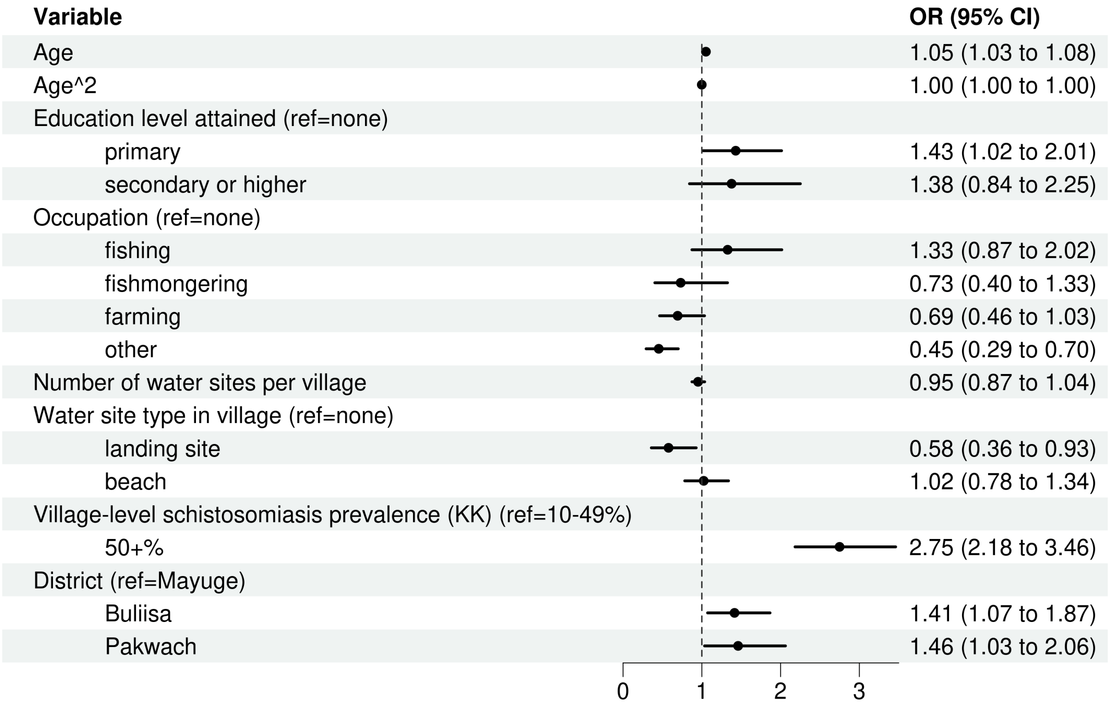

Figure S10: Zero-inflated negative binomial regression model predicting *S. mansoni* infection intensity among all participants (n=2867). Intensity from Kato-Katz, measured in eggs per gram of stool. Standard errors clustered at the household level. A zero inflation test suggested that the predicted number of zeros (1531) was not significantly different from the number of observed zeros (1531), ratio=0.94, at the default tolerance of 0.1 of the `check_zeroinflation` function in the R package `performance`. Abbreviations: OR = odds ratio. CI = confidence interval.

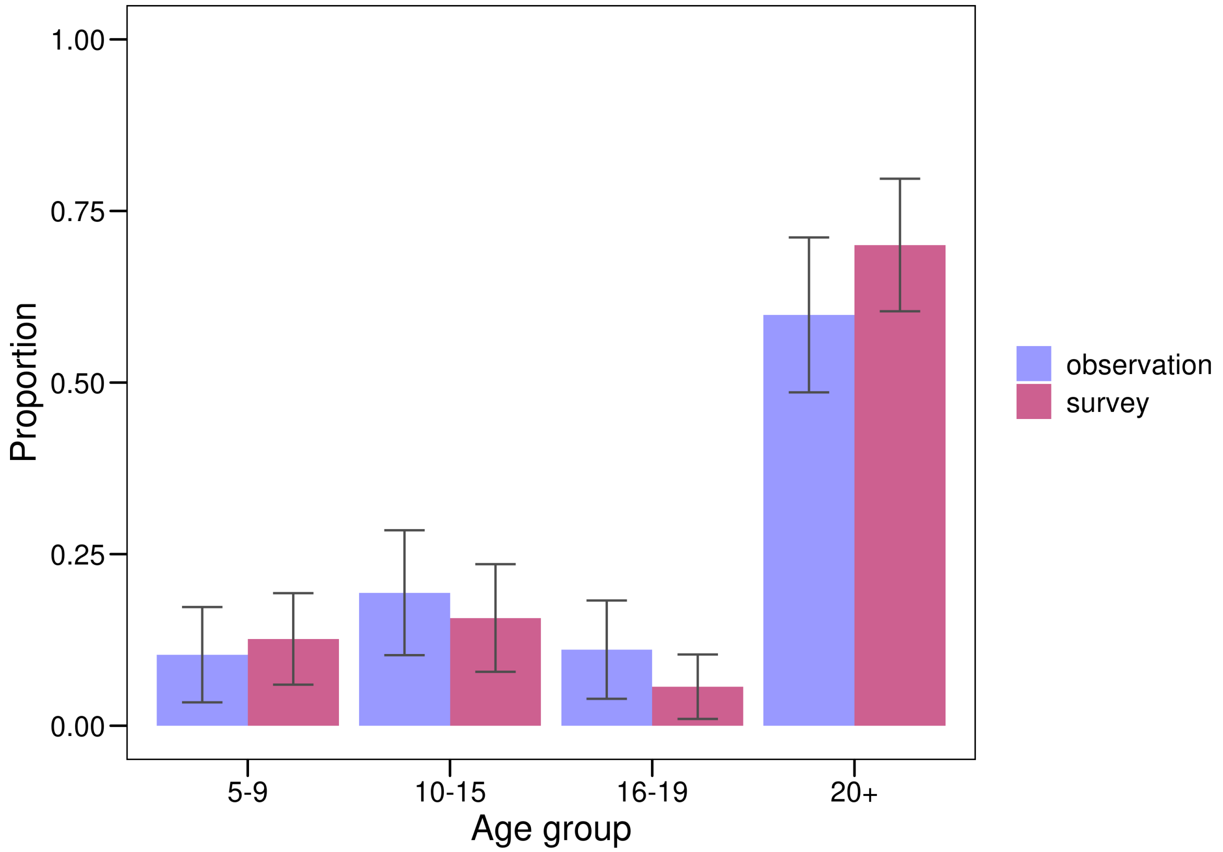

Figure S11: Comparison of age distribution in self-reported data with age distribution of direct observation data in 12 villages with both observation and survey data (survey data n=605 and direct observation n=13,515). Proportions represent the proportion of all water contacts which occur in each age group. Age groups were based on the categories used for MDA treatment and used because direct observation was conducted using these age groups. Whiskers represent 95% confidence intervals obtained using bootstrapping with 1000 repeats.

Variable	OR (95% CI) infection	OR (95% CI) infection reclassified
Age	1.05 (1.03 to 1.08)	1.06 (1.03 to 1.08)
Age^2	1.00 (1.00 to 1.00)	1.00 (1.00 to 1.00)
Education level attained (ref=none)		
primary	1.53 (1.20 to 1.94)	1.40 (1.10 to 1.77)
secondary or higher	0.89 (0.61 to 1.28)	0.74 (0.52 to 1.06)
Occupation (ref=none)		
fishing	1.16 (0.81 to 1.64)	1.01 (0.71 to 1.45)
fishmongering	0.77 (0.48 to 1.22)	0.85 (0.54 to 1.36)
farming	0.62 (0.46 to 0.83)	0.68 (0.51 to 0.91)
other	0.55 (0.39 to 0.77)	0.59 (0.42 to 0.83)
Number of water sites per village	0.88 (0.82 to 0.94)	0.84 (0.78 to 0.90)
Water site type in village (ref=none)		
landing site	0.51 (0.37 to 0.68)	0.37 (0.27 to 0.51)
beach	1.06 (0.86 to 1.32)	1.36 (1.09 to 1.69)
Village-level schistosomiasis prevalence (KK) (ref=10-49%)		
50+%	3.26 (2.73 to 3.90)	2.88 (2.38 to 3.47)
District (ref=Mayuge)		
Buliisa	1.02 (0.81 to 1.28)	1.01 (0.79 to 1.28)
Pakwach	1.15 (0.91 to 1.45)	1.40 (1.12 to 1.77)

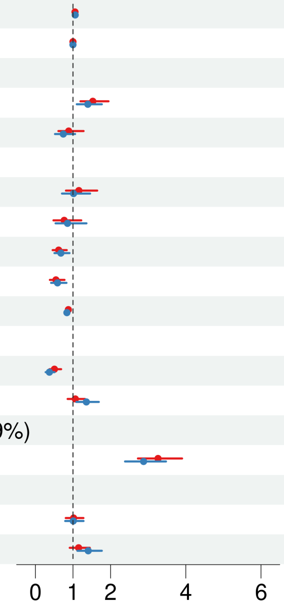

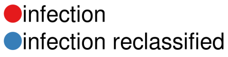

Figure S12: Seperate logistic regression model predicting infection status based on Kato-Katz as well as reclassified Kato-Katz (where participants were recoded as infected whenever Kato-Katz infection status was negative but when the more sensitive POC-CCA diagnostic (positive band 1-3) indicated an infection, n=2867). Standard errors clustered at the household level. Abbreviations: OR = odds ratio. CI = confidence interval.

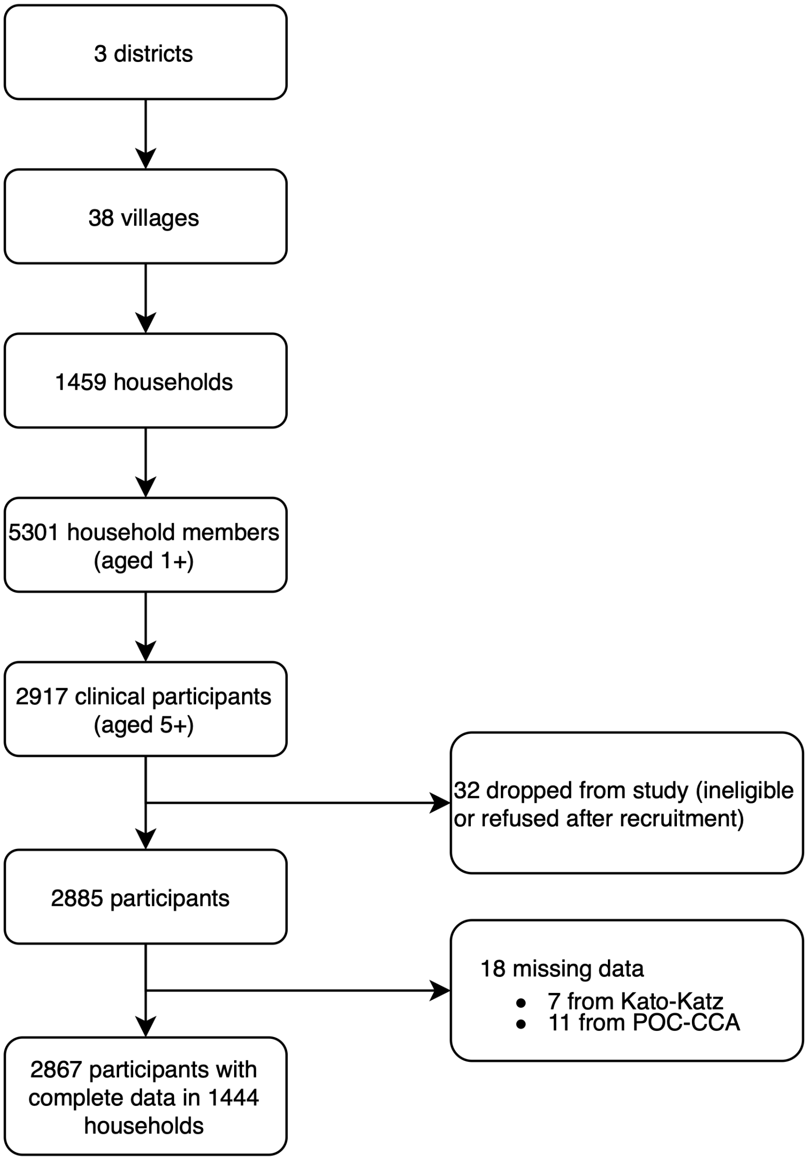

Figure S13: Participant flow diagram

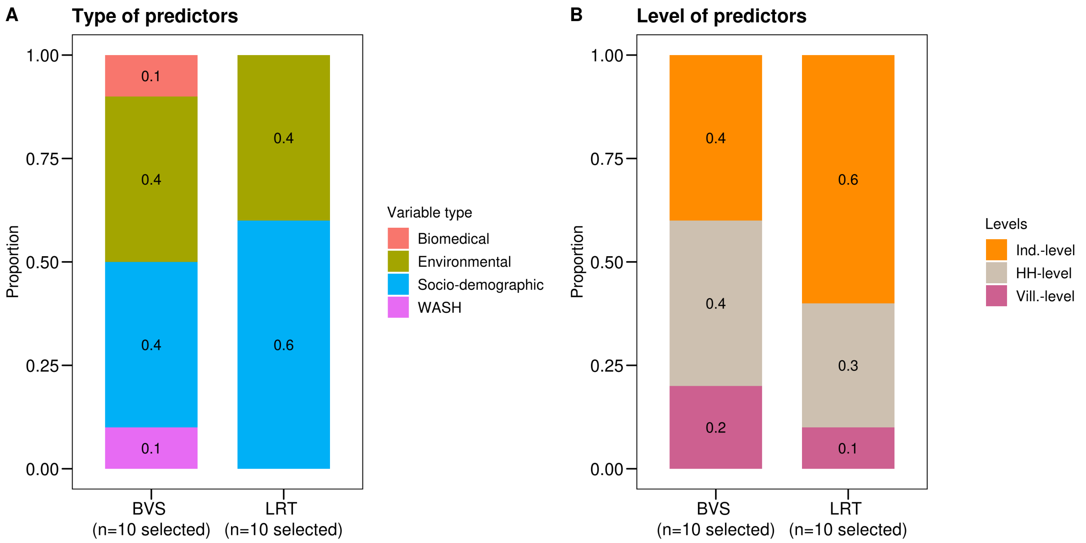

Figure S14: Comparison of variable selection results from BVS and LRTs for predicting water contact. Panel **A** shows the percentage of selected variables from Bayesian variable selection (BVS, n=10 selected variables) and likelihood ratio tests (LRTs, n=10 selected variables) by type of variable. Variables selected using LRTs were solely socio-demographic and environmental variables, while BAS also selected water, sanitation, and hygiene (WASH) and biomedical variables. Among socio-demographic variables, age, age^2, gender, and occupation were consistently selected by BAS and LRTs. Current school enrolment status and educational attainment were only selected by LRT. Among environmental variables, contamination at the closest water site and presence of a water site within the village consistently selected. Distance variables were only selected by LRTs (household distance to closest water site and village centre distance). BVS selected distance to the closest public latrine, the type of site closest to the household, contamination at the closest site, and village-level infection prevalence. No WASH variables were selected via LRT. In BVS, the WASH variables number of latrines per village and proportion of households using an improved drinking water source were selected. Panel **B** compares selected variables from BVS and LRTs by level. In LRTs, 60% of selected variables were individual level, while the same figure was 40% for BAS. The proportion of village-level variables was twice as high in the BVS variable set (20%) compared to the set from LRTs (10%).

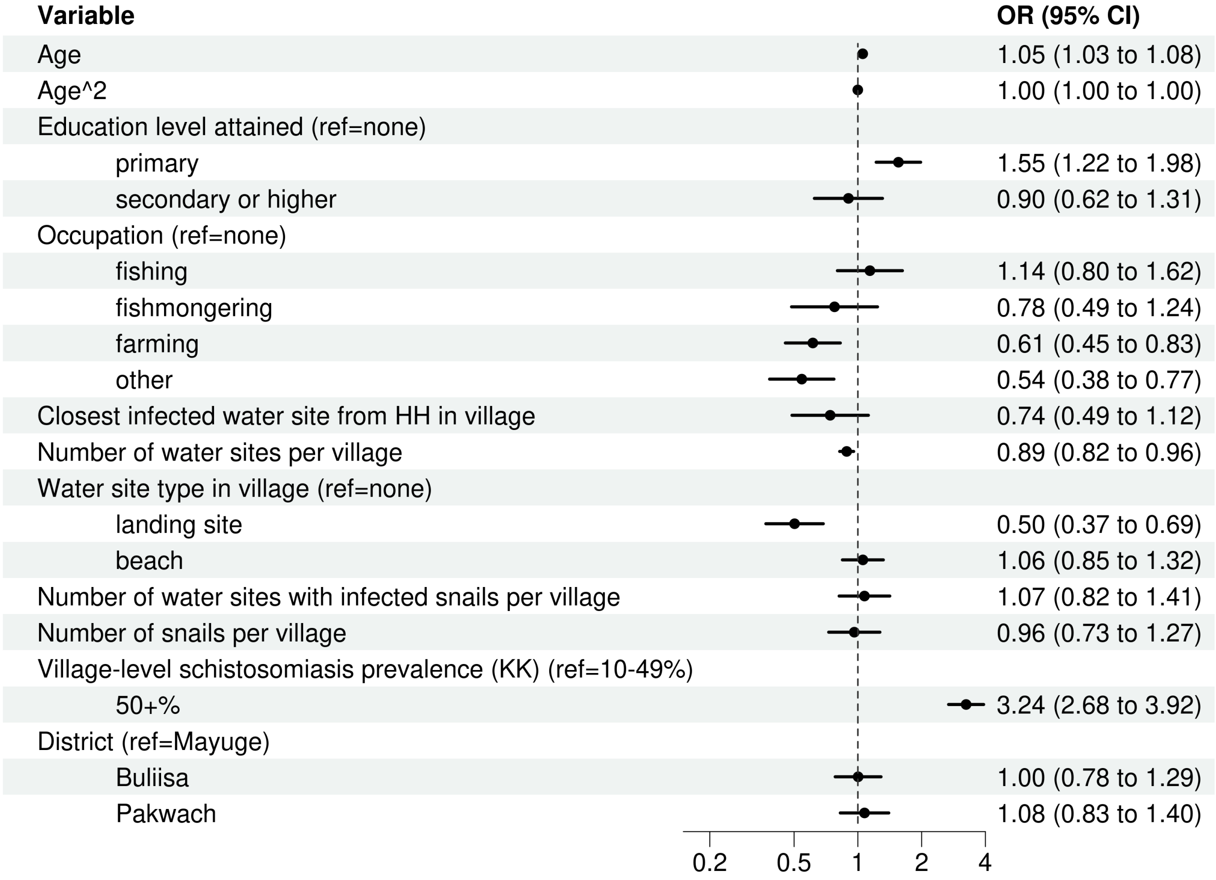

Figure S15: Logistic regression model predicting infection status among all participants using the main predictor variables plus a more extensive set of snail variables selected using Bayesian variable selection, see methods for details (n=2867). Standard errors clustered at the household level. Additional snail variables selected via BVS were distance to the closest water site with infected snails and the number of infected snails at the closest water site. Abbreviations: OR = odds ratio. CI = confidence interval.

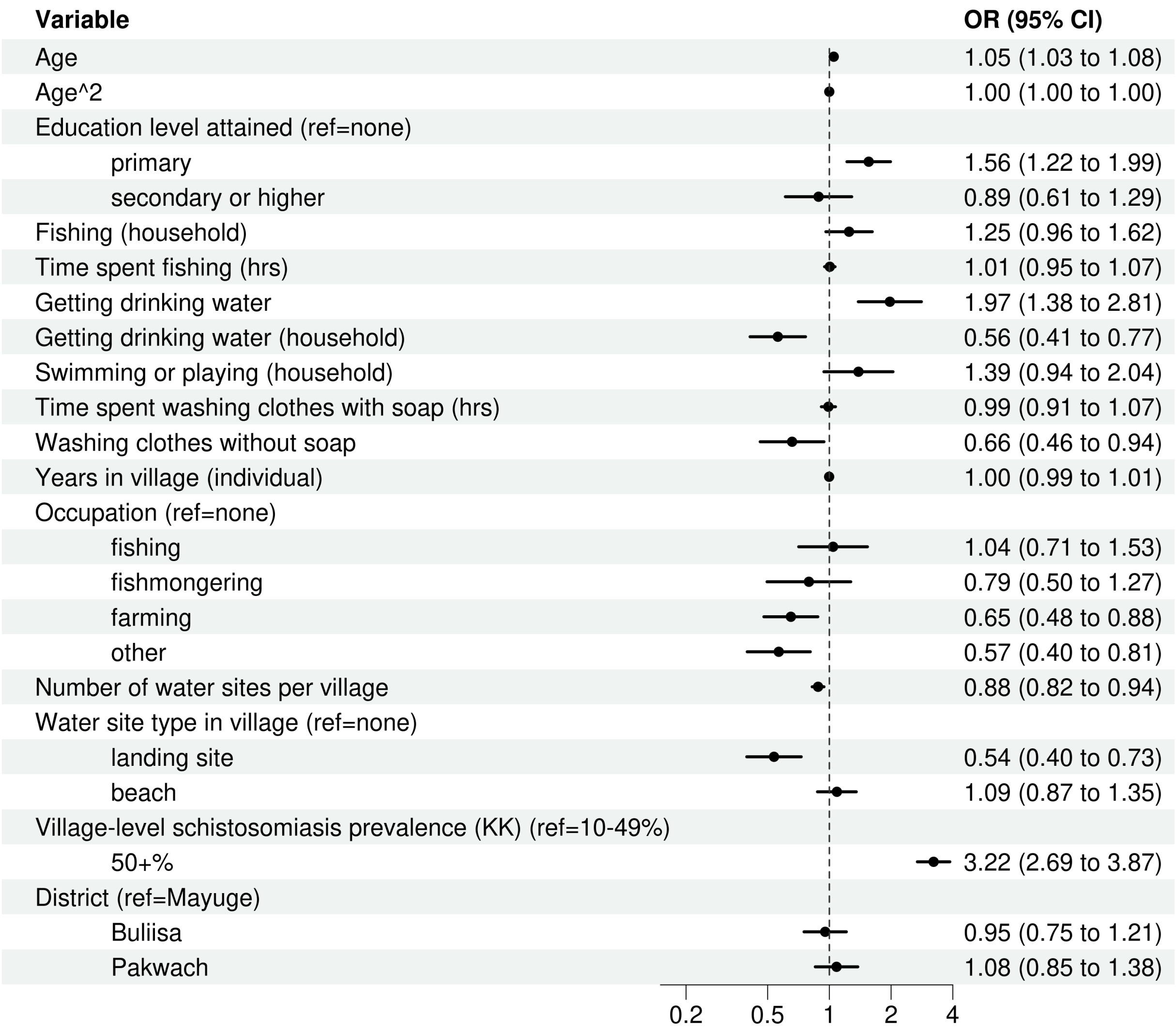

Figure S16: Logistic regression model predicting infection status among all participants using the main predictor variables plus a more extensive set of water contact variables selected using Bayesian variable selection, see methods for details (n=2867). The six additional granular water contact variables selected via BVS were years of residence in the village, time spent on fishing, getting drinking water, household-level swimming or playing, washing clothes with soap, household-level fishing, time spent on washing clothes with soap, and household-level getting drinking water. Abbreviations: OR = odds ratio. CI = confidence interval.
