# Supplementary material for: Current water contact and Schistosoma mansoni infection have distinct determinants: a data-driven population-based study in rural Uganda
Source: Nat Commun. 2024 Nov 8;15:9530. doi: 10.1038/s41467-024-53519-4 (PMC11549081; doi:10.1038/s41467-024-53519-4)
Supplement: Supplementary file 3 — Reporting Summary [file 41467_2024_53519_MOESM3_ESM.pdf]

nature portfolio

Corresponding author(s): Goylette F. Chami

Last updated by author(s): 10/10/2024

Reporting Summary

Nature Portfolio wishes to improve the reproducibility of the work that we publish. This form provides structure for consistency and transparency in reporting. For further information on Nature Portfolio policies, see our Editorial Policies and the Editorial Policy Checklist.

Statistics

For all statistical analyses, confirm that the following items are present in the figure legend, table legend, main text, or Methods section.

n/a	Confirmed	
☐	☒	The exact sample size (n) for each experimental group/condition, given as a discrete number and unit of measurement
☒	☐	A statement on whether measurements were taken from distinct samples or whether the same sample was measured repeatedly
☐	☒	The statistical test(s) used AND whether they are one- or two-sided<br>*Only common tests should be described solely by name; describe more complex techniques in the Methods section.*
☐	☒	A description of all covariates tested
☐	☒	A description of any assumptions or corrections, such as tests of normality and adjustment for multiple comparisons
☐	☒	A full description of the statistical parameters including central tendency (e.g. means) or other basic estimates (e.g. regression coefficient) AND variation (e.g. standard deviation) or associated estimates of uncertainty (e.g. confidence intervals)
☐	☒	For null hypothesis testing, the test statistic (e.g. F, t, r) with confidence intervals, effect sizes, degrees of freedom and P value noted<br>*Give P values as exact values whenever suitable.*
☐	☒	For Bayesian analysis, information on the choice of priors and Markov chain Monte Carlo settings
☐	☒	For hierarchical and complex designs, identification of the appropriate level for tests and full reporting of outcomes
☐	☒	Estimates of effect sizes (e.g. Cohen's d, Pearson's r), indicating how they were calculated

Our web collection on statistics for biologists contains articles on many of the points above.

Software and code

Policy information about availability of computer code

Data collection	Open Data Kit (ODK) was used for data collection (ODK tablet app version v2021.3.1, ODK XLSForm Offline v1.9.0)
Data analysis	R version 4.1.0 was used for data analysis

For manuscripts utilizing custom algorithms or software that are central to the research but not yet described in published literature, software must be made available to editors and reviewers. We strongly encourage code deposition in a community repository (e.g. GitHub). See the Nature Portfolio guidelines for submitting code & software for further information.

Data

Policy information about availability of data

All manuscripts must include a data availability statement. This statement should provide the following information, where applicable:

- Accession codes, unique identifiers, or web links for publicly available datasets
- A description of any restrictions on data availability
- For clinical datasets or third party data, please ensure that the statement adheres to our policy

Data is not publicly available due to data protection and ethics restrictions related to the ongoing nature of the SchistoTrack cohort and easily identifiable nature of the participants. We provide code and demonstration data to rerun our analysis pipeline. We provide all relevant metadata for all variables within the manuscript and supplement, as well as an extensive data dictionary detailing all variable definitions which will be available upon publication.

Research involving human participants, their data, or biological material

Policy information about studies with human participants or human data. See also policy information about sex, gender (identity/presentation), and sexual orientation and race, ethnicity and racism.

Reporting on sex and gender	We exclusively use the term gender, following the definitions laid out in the 'Sex and Gender Equity in Research – SAGER – guidelines' because in this study, information was elicited via self-reports and we sought to understand how gender (shaped by social and cultural norms) influences water contact behaviour. We explicitly focus on gender in our analysis and report multiple breakdowns by gender in this study (see for instance Figures 3, 7 and Supplementary Tables 3-6).
Reporting on race, ethnicity, or other socially relevant groupings	We recorded self-reported data on tribe (Alur, Banyoro, Bagungu, Musiki, Musoga, Jaluo, Mulamongi, Musamya, Muganda, Mudama, Muteso, Other, Mugwere, Lugbara, Munyole, Kakwa, Mukenye, Mugisu, Munyarwanda, Munyankole, Baganda, Mukiga, Madi, Balalo). We also recorded self-reported data on religion (Christian, Muslim, Born-again christian, no religion). These data were recorded as different tribes and religious groups have been found to have different water contact patterns in past research. However, in our analyses these variables were not selected for inclusion in the main models.
Population characteristics	See behavioral & social study design section below
Recruitment	We randomly sampled a total of 1459 households, approximately 40 per village, from village registers or MDA records. All households with at least one child and one adult residing in the village for at least six months of the year were eligible.
Ethics oversight	Data collection and use were reviewed and approved by Oxford Tropical Research Ethics Committee (OxTREC 509-21), Vector Control Division Research Ethics Committee of the Uganda Ministry of Health (VCDREC146), and Uganda National Council of Science and Technology (UNCST HS 1664ES).

Note that full information on the approval of the study protocol must also be provided in the manuscript.

Field-specific reporting

Please select the one below that is the best fit for your research. If you are not sure, read the appropriate sections before making your selection.

☐ Life sciences ☒ Behavioural & social sciences ☐ Ecological, evolutionary & environmental sciences

For a reference copy of the document with all sections, see nature.com/documents/nr-reporting-summary-flat.pdf

Behavioural & social sciences study design

All studies must disclose on these points even when the disclosure is negative.

Study description	Cross-sectional study of 2867 individuals aged 5-90 years in Eastern and Western Uganda. We collected socio-demographics, biomedical information (including schistosome infection status), WASH information, environmental data, and water contact data,
Research sample	Population representative sample of 2867 participants across 40 villages aged 5-90, 1573 females and 1294 males
Sampling strategy	We randomly sampled a total of 1459 households, approximately 40 per village, from village registers or MDA records. All households with at least one child and one adult residing in the village for at least six months of the year were eligible. After obtaining informed consent, questionnaires were administered to obtain socio-demographics, biomedical variables, WASH and environmental variables, and water contact patterns on all household members aged 1+. At the end of the interview, one adult (aged 18+) and one child (aged 5-17) per household were selected for clinical assessments by the household head. All participants were treated for schistosomiasis using praziquantel following the clinical examinations.
Data collection	Household data collection was done by trained surveyors (either from Kampala or locally recruited) using tables and ODK software. Household-level information was collected prior to clinical data collection and participants were unaware of their infection status. All clinical assessments were conducted by technicians/nurses from the district or Kampala in mobile in-the-field labs.
Timing	Data collection took place in January to February 2022 in Pakwach, Buliisa and Mayuge.
Data exclusions	A flow chart detailing any data exclusions is provided in Figure S13. Among 2885 participants, 32 were excluded because of incomplete infection measures.
Non-participation	We initially recruited 2917 participants for clinical assessments but due to refusals and non-participation, the number of participants with at least one clinical sample was 2885.
Randomization	Not applicable as this was an observational study.

Reporting for specific materials, systems and methods

We require information from authors about some types of materials, experimental systems and methods used in many studies. Here, indicate whether each material, system or method listed is relevant to your study. If you are not sure if a list item applies to your research, read the appropriate section before selecting a response.

Materials & experimental systems

n/a	Involved in the study
☒	☐ Antibodies
☒	☐ Eukaryotic cell lines
☒	☐ Palaeontology and archaeology
☒	☐ Animals and other organisms
☐	☒ Clinical data
☒	☐ Dual use research of concern
☒	☐ Plants

Methods

n/a	Involved in the study
☒	☐ ChIP-seq
☒	☐ Flow cytometry
☒	☐ MRI-based neuroimaging

Clinical data

Policy information about clinical studies

All manuscripts should comply with the ICMJEguidelines for publication of clinical research and a completedCONSORT checklist must be included with all submissions.

Clinical trial registration	Not applicable as this was an observational study.
Study protocol	Not applicable as this was an observational study.
Data collection	Data collection took place in January to February 2022 in Pakwach, Buliisa and Mayuge.
Outcomes	Primary exposure outcome was water contact, primary infection outcome was schistosome infection, as indicated by Kato-Katz stool microscopy.

Plants

Seed stocks	Not applicable
Novel plant genotypes	Not applicable
Authentication	Not applicable
